# Successional Change in Phosphorus Stoichiometry Explains the Inverse Relationship between Herbivory and Lupin Density on Mount St. Helens

**DOI:** 10.1371/journal.pone.0007807

**Published:** 2009-11-12

**Authors:** Jennifer L. Apple, Michael Wink, Shannon E. Wills, John G. Bishop

**Affiliations:** 1 School of Biological Sciences, Washington State University, Vancouver, Washington, United States of America; 2 Institut für Pharmazie und Molekulare Biotechnologie, Universität Heidelberg, Heidelberg, Germany; Umea University, Sweden

## Abstract

**Background:**

The average nitrogen-to-phosphorus ratio (N∶P) of insect herbivores is less than that of leaves, suggesting that P may mediate plant-insect interactions more often than appreciated. We investigated whether succession-related heterogeneity in N and P stoichiometry influences herbivore performance on N-fixing lupin (*Lupinus lepidus*) colonizing primary successional volcanic surfaces, where the abundances of several specialist lepidopteran herbivores are inversely related to lupin density and are known to alter lupin colonization dynamics. We examined larval performance in response to leaf nutritional characteristics using gelechiid and pyralid leaf-tiers, and a noctuid leaf-cutter.

**Methodology/Principal Findings:**

We conducted four studies. First, growth of larvae raised on wild-collected leaves responded positively to leaf %P and negatively to leaf carbon (%C), but there was no effect of %N or quinolizidine alkaloids (QAs). Noctuid survival was also positively related to %P. Second, we raised gelechiid larvae on greenhouse-grown lupins with factorial manipulation of competitors and soil N and P. In the presence of competition, larval mass was highest at intermediate leaf N∶P and high %P. Third, survival of gelechiid larvae placed on lupins in high-density patches was greater when plant competitors were removed than on controls. Fourth, surveys of field-collected leaves in 2000, 2002, and 2003 indicated that both %P and %N were generally greater in plants from low-density areas. QAs in plants from low-density areas were equal to or higher than QAs in high-density areas.

**Conclusions/Significance:**

Our results demonstrate that declines in lupin P content under competitive conditions are associated with decreased larval growth and survival sufficient to cause the observed negative relationship between herbivore abundance and host density. The results support the theoretical finding that declines in stoichiometric resource quality (caused here by succession) have the potential to cause a decrease in consumer abundance despite very dense quantities of the resource.

## Introduction

Predicting the spatial and temporal dynamics of consumer populations as a function of macronutrient and energy resources has a long history in theoretical and empirical ecology. Response to nitrogen (N) has been a particular focus for understanding the dynamics of terrestrial insect herbivores [1], [2], [3], [4], [5], and provides the mechanistic basis for some proposals that predict herbivore feeding patterns [4], [6]. For example, as predicted by one version of the plant stress hypothesis [7], boring insect guilds respond positively to drought stress, as do phloem-feeding insects when drought stress is intermittent [8], effects that occur because of enhanced N availability in drought-stressed plants. Likewise, tests for bottom-up control of terrestrial herbivore populations typically manipulate soil N availability, sometimes with dramatic enhancement of herbivore densities [9], [10], [11], [12], [13], [14], [15].

Studies of terrestrial arthropod N limitation have frequently considered N not only in isolation, but in relation to quantities of plant defensive chemicals and carbohydrates. Nevertheless, explicit simultaneous consideration of multiple nutritional requirements (especially C, N, and P), known as ecological stoichiometry, has only recently been applied to the interaction of terrestrial autotrophs and their consumers. In contrast to classical arguments in favor of N limitation, stoichiometric analyses suggest that plant phosphorus (P) content may be an important but unrecognized limiting nutrient for many terrestrial insect herbivores [16], [17]. This proposal arises from comparison of the nutrient supply ratio (i.e. autotroph N∶P) to the demand ratio dictated by the relatively homeostatic physiology of arthropods: the N∶P in plant leaves is, on average, about 21% greater than the N∶P of the average terrestrial insect herbivore [17]. Similar imbalances in consumer-resource N∶P ratios are found in aquatic systems where invertebrate heterotrophs have more often proved to be P-limited [17]. However, it is unclear under what conditions P limitation should be expected, since P-limitation is not determined by N∶P stoichiometry alone. For example, even when the imbalance in consumer-resource N∶P appears to favor P limitation, protein-precipitating defensive chemicals (i.e. tannins) or other forms of defense could promote N limitation [18], [19], [20].

Relatively few studies have sought evidence for P sensitivity in terrestrial insect herbivores. In a review of how plant nutrient stress impacts insect herbivores, Waring and Cobb [21] identified several fertilization experiments that showed positive responses of herbivores to leaf P content. A handful of other studies suggest P limitation or P sensitivity of terrestrial insect herbivores [2], [22], [23], [24], [25], [26], [27] or have examined the importance of P in relation to other elements [3], [28], [29]. Nevertheless, the frequency and conditions under which heterogeneity in P availability affects individual fitness or population growth of terrestrial insect herbivores are relatively unexplored [30].

In this paper we evaluate whether succession-related patterns in plant nutritional and defensive chemistry underlie a remarkable pattern of spatially structured herbivory exhibited by several guilds of lepidopteran herbivores feeding on alpine lupin (*Lupinus lepidus* var. *lobbii*), in which herbivores attack plants in low-density regions of the expanding lupin population while not damaging much denser “core” regions [31]. Herbivore attack strongly impacts the demography and rate of spatial spread at low-density margins of core patches and in the low-density “matrix” into which lupins are colonizing [31], [32], [33], [34]. (Photographs of representative site types are available in Appendix S1.) Because lupin has strong facilitative effects on soil development [35] and on other plant species [36], [37], [38] at Mount St. Helens, these herbivores substantially impact the pace and pattern of community assembly.

In this paper, we present results from three experiments that examine whether the nutrient content of lupin leaves may explain differences in larval abundance between high-density core areas and low-density margin and matrix areas. In the first experiment, we quantified larval growth (and where possible, mortality) of three moth species, representing two leaf feeding guilds, on lupin collected from the center of a patch comprising the core region (hereafter “center”) and the margins of that patch (hereafter “margin”). Because we hypothesize that plant competition for soil resources may be an important determinant of leaf nutritional quality, we examined the response of one leaf-tying species to N and P fertilization and host competitive environment in separate field and greenhouse experiments. We interpret these results in the context of a survey comparing lupin leaf nutritional characteristics between high- and low-density areas. In addition to the fact that N is less likely to be limiting to herbivores of N-fixing plants, several observations led us to hypothesize that P stoichiometry could underlie this spatial pattern: 1) lupins from high-density sites contain less P than those from low-density [39]; 2) lupins at high-density sites are P-limited [40], [41]; 3) N∶P of root-boring and leaf-tying larvae is less than that of lupin tissues from high-density patches but similar to that of tissue from low-density patches [31]; and 4) the abundance of orthopterans responds strongly to P addition at these sites [41].

## Materials and Methods

On Mount St. Helens' Pumice Plain (∼1200 m elevation), adults of the leaf-tying/leaf-mining caterpillars *Filatima loowita* (Lepidoptera: Gelechiidae; [42]; misidentified as *Chionodes spp.* in Bishop [34]) and *Staudingeria albipenella* (Lepidoptera: Pyralidae) mate and oviposit in early June through mid-July, and larvae are active in July and August. Late instar larvae overwinter and pupate in spring. The two species (referred to hereafter as “gelechiid”, “pyralid”, or collectively as “leaf-tiers”) have similar feeding habits and produce indistinguishable damage patterns. As early instars, they mine individual leaflets, while later instars tie leaflets together into silken feeding tubes; individual plants (up to 40 cm diameter and 10 cm tall) may host dozens of larvae. *Euxoa extranea* (Lepidoptera: Noctuidae) (hereafter, *Euxoa*) is an external leaf feeder that mates and oviposits from mid-July until late August. Larvae develop through the fourth or fifth instar before winter diapause, then re-emerge and feed in early summer, passing through 7–8 instars. At Mount St. Helens, all three species appear to feed exclusively on *L. lepidus*, avoiding even adjacent *L. latifolius*. Photos of the species and experiments are provided in Appendix S1. All three species were used for an experiment examining growth on wild-collected leaves, while the gelechiid was used for two additional feeding experiments.

### Performance on Wild-Collected Leaves

Adult *Euxoa* were trapped on 29 July 2003 and allowed to mate and oviposit in cages. Sixty-six first-instar larvae were randomly assigned to each diet treatment (center vs. margin, *N* = 132) and placed in 0.75-oz plastic condiment cups on 21 August ( =  day 1). Fresh lupin shoots were field-collected from a single area of high-density lupin (“center”) and the nearby (<100 m) low-density margins (“margin”) every 1–2 wks and stored at 4°C. See Appendix S1 for photographs of representative margin and center areas. Caterpillars were fed fresh leaves, in excess, every four days and were weighed every 3–7 days starting at day 10. Larvae were maintained in a growth chamber with the following settings: days 1–36: 16-hour day, diurnal range 10°C–25°C, mean = 17.1°C; days 36–66: 10-hour day, diurnal range 7°C–24°C, mean = 14.6°C. Larvae were sacrificed for nutrient quantification on day 63 (in the 6^th^ or 7^th^ instar), slightly later than the onset of winter diapause.

For the gelechiid feeding trial 124 first-instar larvae were reared in pairs in 62 8-oz plastic deli containers. For the pyralid trial, 300 first-instar larvae were reared in groups of three in 100 plastic containers. All larvae were collected on the Pumice Plain in early July 2003. Half of the containers received a vegetative shoot of lupin from the margin area and half received a shoot from the center. Lupin shoots were inserted into tubes of water (1.5 ml Eppendorf tubes). Lupin was collected and stored as for *Euxoa*. Larvae were maintained in a growth chamber set for a 16-hour day (diurnal temperature ranging 10°C–25°C, mean = 17.1°C). They were weighed on days 19 and 35 (gelechiids) and on days 21 and 38 (pyralids).

Comparisons between sites yielded no consistent differences. Therefore, we focused our analysis on the effects of leaf nutrient content. To examine the relationship between nutrients and larval growth, we divided the *Euxoa* feeding trials into five consecutive intervals that corresponded to batches of food collected on different dates, and leaf-tier trials into two intervals. For *Euxoa*, separate relative growth rates (RGR) were calculated over each interval for each larva as the slope of the regression of ln (fresh mass) on length of the interval in days. Regressions involved three to four measurements of mass in an interval, thereby dampening any effects of molting on mass, and avoiding some of the issues related to analysis of ratio-based measures of growth rate [43], [44]. We took the average individual mass (0.00015 g) of first instar *Euxoa* weighed *en masse* as the initial mass for all larvae. To further ensure against spurious correlations related to ratio-based variables [43], [44], we repeated all analyses using larval mass as the dependent variable. Because leaf-tying larvae were only weighed twice (to minimize disruption of their silken feeding shelters), we calculated individual RGR as [ln (final mass) – ln(initial mass)]/(number of days in interval) over two intervals (units are g g^−1^ d^−1^, which simplifies to d^−1^). As with *Euxoa*, we estimated initial mass as the mean mass of first-instar larvae.

Plant material for feeding trials was collected from at least 20 plants per date and usually consisted of shoots with 5–10 leaves at various stages of development. Only shoots lacking insect damage were collected, but during the August 2003 *Euxoa* experiment, nearly all margin plants in the food collection site had damaged portions and thus collection was probably biased toward less palatable material.

Carbon (C) and N content of ground samples were measured on a Perkin-Elmer 2400 CHN analyzer and P was quantified using persulfate digestion and ascorbate-molybdate colorimetry as described previously (Fagan et al. 2004). Quinolizidine alkaloid (QA) profiles were measured as described in Appendix S2. The relationship between elemental ratios, density, and date was analyzed with multiple regression on untransformed data.

To examine the relationship between leaf characteristics and larval growth, we regressed RGR on the corresponding mass percentages of leaf nutrients (%C, %N, and %P) and total QA concentration. Because leaf data were collected by food batch (species × diet source × time interval) rather than by larva, our analysis is focused on mean RGR of larvae feeding on each diet in each food interval, yielding a total of 18 data points (five *Euxoa* intervals × two diets + two leaf-tier intervals × two species × two diets), each representing 15–86 larvae. For the leaf-tying caterpillars, intervals spanned multiple food batches, so leaf data were averaged (weighted by time fed on a batch) for each interval. Because the mean RGR was estimated using the same animals across multiple time intervals, we accounted for repeated measures at the group level by nesting interval within each species × diet source combination in a mixed-effects model [R model: lme(rgr ∼ alkaloids + P + N + C, random =  ∼ interval | group_id), where group_id  =  species × diet source combination]. By nesting interval within group identity we also control for differences in rearing temperature among intervals, and for the observation that larval mass-specific growth rate [45] and leaf nutrient content may decline as development progresses through the growing season. Model simplification was performed by deleting the least significant term and comparing the log likelihoods of the nested models using ANOVA [46]. We also regressed RGR on atomic ratios (C∶N, C∶P, and N∶P), using the same mixed-effect model, to allow comparison of our results to the rapidly growing literature relating nutrient stoichiometry to consumer growth. Results for atomic ratios are presented in Appendix S3 and are similar to those involving mass percentages. As an alternative to analyzing RGR, we also analyzed mean mass and individual mass as the response variable using a mixed effects model with interval date and larval identity as random effects variables.

To better understand whether differences in growth between larvae feeding on different diets during identical time intervals were attributable to differences in leaf characteristics, we calculated the difference in RGR between center and margin for each interval, and regressed this on the difference in leaf characteristics calculated for each time interval. We accounted for repeated measures at the group level by regressing RGR on group identity, then using the residuals of this regression to calculate RGR_center_ - RGR_margin_.

### Greenhouse Fertilization and Competition Experiment

*Filatima* females collected from the field on June 18, 2003 were allowed to oviposit on potted lupin plants. On July 1 ( = day 1), eggs were redistributed so that 12 greenhouse grown plants from each of 8 treatment combinations had 1–3 eggs. The number of larvae per plant was later reduced to 1. Plant treatments were in a two-way factorial design with competition (presence or absence of the grass *Deschampsia caespitosa*) and N or P fertilization (top-water application of control, N, P, or N + P fertilizer solutions) as the main treatments. We applied fertilizer once near the beginning of the experiment. All plants received 100 ml of 5% Hoagland's solution minus the N and P components. N addition treatments received N at a rate of 3.9 mg/kg; all non-N plants were fertilized with N at 5% of this level. P addition treatments received P at a rate of 6 mg/kg soil; all non-P plants received P at 2.5% of this level. N and P application rates mimicked the highest N and P concentrations observed on the Pumice Plain. Plants were 7 months old (and at a typical stage attacked by leaf-tiers) at egg placement. To prevent escape, clear plastic containers with holes in the top were affixed to each pot.

On days 22 and 43 after placement of eggs, we removed larvae and weighed them. Larvae reside in webbed retreats, whose disruption for more frequent weighing would have compromised the experiment. Because of escape and mortality, only 43 larvae survived until day 43. After the second weighing larvae were starved for 3 days and dried at 60°C. Leaf samples from each host plant were collected at day 43 and dried at 60°C. Leaves and larvae were ground and analyzed for C, N, and P content as described above. Analyses were conducted on final dry mass at day 46 and on RGR (calculated as [ln(2^nd^ weight) − ln(1^st^ weight)]/21 days]. Treatment effects on mass and RGR were compared with ANOVA and the relationship of mass to leaf %C, %N, %P, and N∶P was examined using regression. Nutrient measurements and RGR were normally distributed, while larval dry mass at day 46 was squared to meet regression assumptions.

### Field Fertilization and Competition Experiment

To test further the effects of competition and P addition on lupin palatability, we manipulated P availability and competitors in high-density center sites that received P or not beginning in 2003. These plots, including P addition rates, are described in Gill et al. (2006) and Bishop et al. (unpublished manuscript). Twelve ∼equal-sized plants within each P addition and control plot were selected for the removal experiment, of which six had all competing plants within 10 cm removed in 2003 by cutting plants off at ground level (see Appendix S1 for photograph). P was again added and removal zones maintained in June 2004. First instar gelechiid larvae were obtained from matrix and margin plants in late June 2004. On July 7, six larvae were placed on 4 plants in each of the control, removal, and P addition + removal treatments at all four sites, for a total of 48 plants and 288 larvae. Six larvae/plant were used because plants typically host multiple larvae, and preliminary experiments led us to expect high predation rates on larvae, which we did not attempt to control. The P addition without removal treatment was omitted because of a shortage of larvae. Because larvae are extremely difficult to re-capture in the field (owing to their underground retreats), we measured proportion of each plant consumed at day 21 as an index of larval growth and survival. Because of the much faster consumption rate of multiple larvae and later instars, % damage reflects growth and survival rather than compensatory feeding. Damage as a function of treatment (3 levels) was analyzed using a generalized linear model with mixed effects, utilizing the glmmPQL command in the MASS package (R 2.8.1, R Foundation for Statistical Computing, 2008). A quasi-poisson distribution was used to account for poisson-distributed data with overdispersion, and data were grouped within site, which was included as a random effect.

### Natural Variation in Leaf Nutrient and Alkaloid Content

To understand the likely consequences of larval performance as a function of leaf nutrient content for the spatial distribution of herbivores, we characterized the nutrient content of plants from a wider sample of high- and low-density patches. Plant material was collected from throughout center and low-density matrix and margin areas between mid-June and late September in 2002 and 2003. In 2002, low-density matrix and margin sites were combined, whereas in 2003, margin sites were tracked separately. Shoots were combined into a bulk sample for each density × date combination, and analyses were performed on a subsample of each bulk sample. C, N, P, and QAs were quantified as described above.

## Results

### Performance on Wild-Collected Leaves

To test whether natural variation in lupin nutritional value may affect the spatial distribution of herbivores via an effect on larval performance, we raised larvae of all three species on leaves collected from patches of different densities (center vs. margin). However, comparisons of growth on center vs. margin material were inconsistent, with higher performance on margin material in some intervals, on center material in others, and no difference in some (results not shown). We therefore focus analyses on the relationship of growth and mortality to leaf nutritional characteristics.

**Figure 1:**
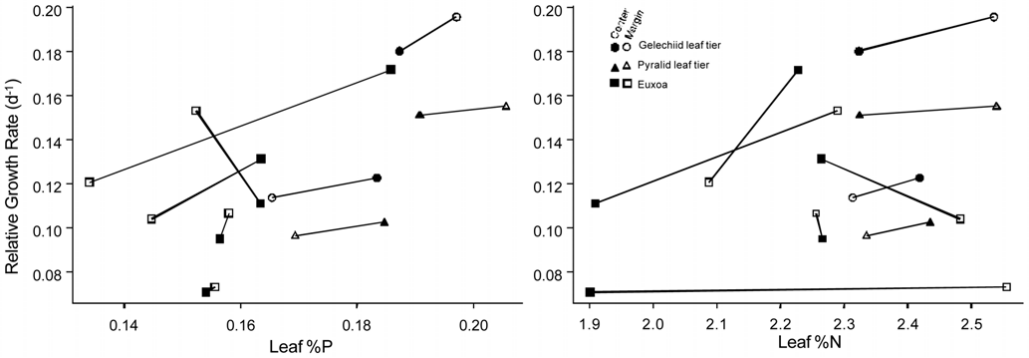
RGR is related to %P in wild-collected leaves. RGR (d^−1^) is closely related to leaf % P (a) but not leaf %N (b). Each point represents the mean of 15–86 individual larvae fed a particular batch of food. Groups of larvae feeding on different diets during the same time interval are connected by lines. Filled symbols represent feeding on leaves from high-density center areas.

Multiple regression of RGR on leaf characteristics for the combined guilds revealed significant effects of %P and %C but not of %N or total QAs (Figs 1a, 1b; Table 1, % denotes mass percentage). As expected, effects of C∶P and C∶N were nearly identical to those of %P and %N (Appendix S3; all ratios are molar ratios). Plant %P had a positive relationship with caterpillar RGR (*P* = 0.008) while %C had a negative effect (*P* = 0.009; Table 1). Regressions for *Euxoa* alone showed marginally significant effects of %P and %C (*N* = 10, *P* = 0.08 and *P* = 0.07, respectively) but not %N or alkaloids (Appendix S3). Regression analysis of *Euxoa* mortality (leaf-tier mortality was not available) also revealed a significant positive effect of leaf %P on survival, but not of %N, N∶P, or alkaloids (for %P: *P* = 0.015, *r* ^2^ = 0.54; See Appendix S4). In a preliminary experiment in 2002, Euxoa mortality was also higher on center plants than on margin plants (Appendix S5). For leaf-tiers analyzed alone, regressions revealed a significant negative effect of N∶P (N = 8, *P* = 0.010) and marginally significant effects of %P and C∶P (*P* = 0.08) (Appendix S3). Analysis of ln(mass) (with date and larval identity as covariates) for the combined guilds yielded similar results to those for RGR (Appendix S4). Analyses of mass for separate guilds were not significant for leaf-tiers, while for *Euxoa* there was a significant positive effect of %P (*P* = 0.03, Appendix S4).

**Table 1:** Regression analysis of nutritional effects on herbivore RGR (d^−1^) or mass (mg).

	Effect	Mean	Coefficient	*P*-value
**WILD LEAF EXPERIMENT**				
***Euxoa*** ** & leaf-tier RGR** a, b, c	%P	0.17	0.94	0.008
*N* = 18, *DF* = 1, 9	%C	44.7	−0.02	0.009
	Alkaloids	0.0044	−1.45	0.106
**RGR difference (center – margin) (** ***Euxoa*** ** & leaf-tiers)** d	Alkaloid Difference	−0.0025	−6.75	0.054
*r* ^2^ = 0.72, *N* = 9, *F* = 7.8, *DF* = 2,6, *P* = 0.021	%P Difference	0.0097	0.77	0.037
**GREENHOUSE EXPERIMENT**				
**Gelechiid dry mass** (mg) **with competitors** e	N∶P	37.5	2.40	<0.0001
*r* ^2^ = 0.73, *N* = 23, *F* = 17.0, *DF* = 3,19, *P*<0.001	N∶P^2^		−0.027	<0.0001
	%P	0.11	58.2	0.002

Sources were either high-density center or the low-density matrix (in 2002 the matrix sample included some samples from the margin). Regression coefficients are shown in the Day and Center vs. Matrix columns.

a Linear Mixed Effects model with time interval and group identity (*species* × *diet source*) as random effects. No r^2^ is available for a mixed effects model. See Materials and Methods for model.

b Mean RGR (d^−1^): *Euxoa*: 0.114, leaf-tiers: 0.142, combined: 0.128.

c Dropping %N had no effect on model fit (model comparison by ANOVA: p = 0.668).

d RGR was first regressed on group identity to account for repeated measures, then the residuals were used to calculate RGR_center_ − RGR_margin_. Dropping %N had no effect on model fit (ANOVA: F = 1.17, DF = 1, p = 0.327).

e Dry mass was squared to meet regression assumptions. Mean dry mass  = 8.7 mg (with competitors). Molar ratio was used for N∶P, and %P had a low correlation to N∶P (*r* = −0.11). Dropping any of the terms in this model dramatically increased the AIC.

Pairwise differences in RGR between center and margin during each feeding period were compared as another way to control for the expected declines in larval mass-specific growth rate and leaf nutrient content through the growing season. Differences between center and margin RGR were strongly correlated with center-margin differences in tissue %P (analysis of all three species; partial *r* ^2^ = 0.61 for RGR), but only weakly correlated with differences in %N (Table 1). Differences in alkaloid content also explained significant variation in RGR in the pairwise analysis (partial *r* ^2^ = 0.34) but not in most other analyses.

### Greenhouse Fertilization and Competition Experiment

**Figure 2:**
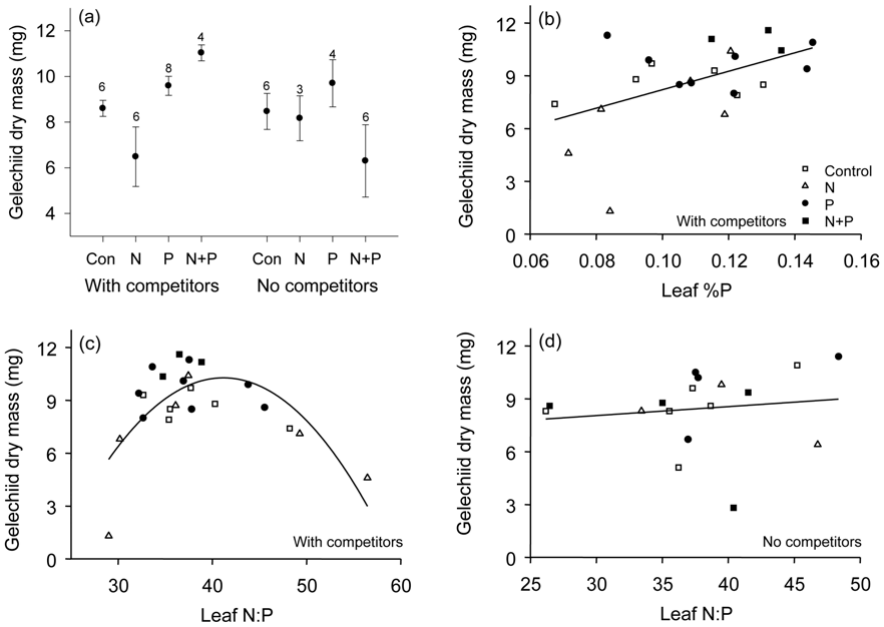
Greenhouse fertilization and competition treatments. a) Treatment effects on larval dry mass (mean ± SE, n is shown above each point) at 46 days. There are significant effects of P and N × P × competition. Panels b–d: The effects of leaf (N∶P)^2^ and %P on larval dry mass at 46 days are highly significant (*P*<0.0001; *P* = 0.002; Table 1) with grass competitors (b,c) but not without competitors (d). The legend in (b) applies to (c) and (d).

As a second test of whether leaf nutritional value may affect the spatial distribution of herbivores via an effect on larval performance, we manipulated lupin leaf quality through altering competition and soil N or P. There was a significant positive effect of P addition on larval RGR and final dry mass of gelechiid leaf-tiers (Fig. 2a; RGR: *P* = 0.022; *F* = 5.9, *DF* = 1,29; dry mass: *P* = 0.023; *F* = 5.0, *DF* = 1,33), and for dry mass there was a significant interaction between plant P, N, and competition (*P* = 0.025, *F* = 5.5, *DF* = 1,33). However, treatments had little effect on leaf nutrient content (%N, %P, C∶N, C∶P, or N∶P), except in the presence of competition, where P addition increased leaf %P and %N (%P: *P* = 0.02, *F* = 6.1; %N: *P* = 0.03, *F* = 5.2; *DF* = 1, 21). Regression of gelechiid RGR and dry mass on leaf nutrient concentration revealed a positive effect of leaf %P and a quadratic relationship to plant N:P for larvae feeding on lupins in competition with grasses, but not for plants without competitors (Figs. 2b–d; Table 1). The quadratic term reflects a hump-shaped relationship between larval mass and plant N∶P, with larvae growing especially slowly at very high N∶P, but also at low N∶P.

### Field Fertilization and Competition Experiment

**Figure 3:**
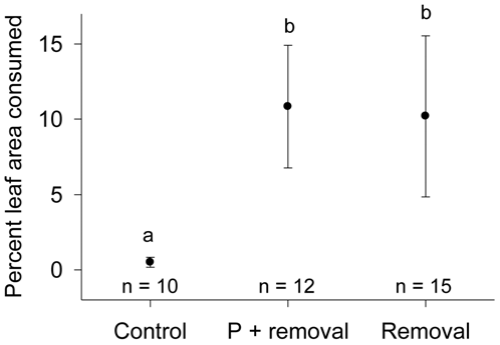
Field fertilization and competitor removal experiment. Percent leaf area consumed by gelechiid leaf-tiers (mean ± SE) is used as an index of survival and growth on plants with competitors removed the previous summer and competitors removed + P fertilization. Survival is almost 0 on plants with neighbors. Treatments not sharing a letter (above error bars) are significantly different (*P* = 0.01).

We tested whether removal of plant competitors or addition of P improved larval performance on plants in the field at the center of core patches. There was a strong positive effect of competitor removal on larval survival: of 37 plants that were re-located, 16 hosted surviving larvae, and fourteen of those with survivors were plants whose competitors had been removed (Competitor removal only: *N* = 6 plants; removal + P addition: *N* = 8 plants; 11 plants were lost because markers were overgrown or damaged by elk; re-located plants were distributed across all patch x treatment combinations, and plants with surviving larvae were evenly distributed across sites). Because the larvae often hide in the soil or in intricate woven tunnels, they are difficult to retrieve in the field. Therefore, we quantified the proportion of the plant tied and consumed as an indicator of larval survival and growth. Inspection of plants with higher damage suggested a greater number of active larvae. Leaf damage was significantly greater in both the P addition + removal treatment and with removal alone than in the controls (generalized linear regression mixed effects model, with plot as a random effect, *DF* = 31; t = 2.52, *P* = 0.017; and t = 2.00, *P* = 0.054) (Fig. 3). The two treatments did not differ from each other.

### Natural Variation in Leaf Nutrient and Alkaloid Content

**Figure 4:**
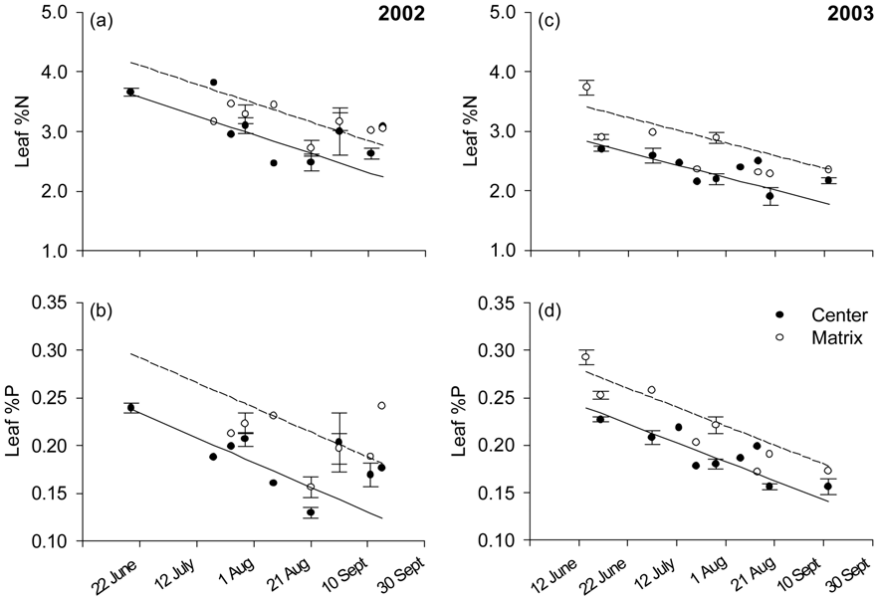
Leaf nutrient concentrations over the 2002 (a and c) and 2003 (b and d) growing seasons, by location. Each point is from a homogenized bulk collection sampled from many plants. Points with error bars represent the mean (± SE) of samples from multiple sites on that date (data from each sample were used in the regression analysis). Matrix refers to low-density areas being colonized by lupins. Plants from matrix areas were much richer in both %N and %P in both years (*P*<0.0002; see Table 2). Least squares fits are shown by solid (center) and dotted (matrix) lines.

To understand the consequence of the effects of leaf P on larval performance for the spatial distribution of herbivores, we characterized the nutrient content and QAs of plants from a wider sample of high- and low-density patches. N and P concentrations were highest on the earliest dates measured (May), and became more dilute as shoots matured (Fig. 4, Table 2); Over most dates in both 2002 and 2003 leaf %P and %N were significantly higher in low-density matrix and margin plants than in center plants, while N∶P was lower in matrix but not margin (Table 2). (In 2002, we did not distinguish between “matrix” and “margin”; samples were combined for these two types of site). The same pattern was also documented in 2000 [39] and, for N, in an independent sample from 2002 (Gill et al. 2006). However, on several dates in 2003 (Fig. 1) caterpillar food from margin areas was lower in N and P content than food from center areas (also see Table 2), whereas non-food collections around these dates display the typical pattern (Fig. 4). Because damage was very high in August 2003 in the pre-defined food source area, the remaining undamaged material available as a food source was probably relatively unpalatable low-nutrient foliage.

**Table 2:** Regression analyses of lupin leaf tissue nutrient and alkaloid content on date and source.

		Day	Center vs. Matrix^a^	*r* ^2^	*N*
**2002**	%N	−0.0158****	0.2662***	0.65	38
	%P	−0.0013****	0.0290****	0.64	36
	N∶P	0.070*	−2.38*	0.14	36
	QAs	0.001**	0.0028*	0.52	22
**2003**	%N	−0.0106****	0.2904****	0.66	56
	%P	−0.0010****	0.0188****	0.76	56
	N∶P	0.027*	0.630*	0.15	56
	QAs	0.99	0.0011	0.12	24

Sources were either high-density center or the low-density matrix (in 2002 the matrix sample included some samples from the margin). Regression coefficients are shown in the Day and Center vs. Matrix columns.

a Negative coefficient indicates a higher value in center plants.

P-values: * = p≤0.05, ** = p≤0.01, *** = p≤0.001, **** = p≤0.0001

As with other lupins, the alkaloid profile of *L. lepidus* is dominated by quinolizidine alkaloids (QAs). The major QAs observed were 3-hydroxylupanine and tigloyl- and angeloyl esters of hydroxylupanines. Minor alkaloids included the pyrrolidine alkaloid ammodendrine, as well as lusitanine, dihydroxylupanine and its angeloyl and tigloyl esters. Total alkaloid content ranged from undetectable to a high of 0.02% of dry mass (Appendix S2). Alkaloids in plants where herbivores had been excluded with pesticide were near 0, indicating that alkaloid production is induced by insect feeding (Appendix S2). However, with only five herbivore exclusion samples, this difference was only marginally significant (repeated-measures ANOVA, *P* = 0.08, *DF* = 1,8, *F* = 4.0). The lack of detectable alkaloids in many leaf samples indicates that harvesting shoots for feeding experiments did not substantially induce alkaloid production. Maximum alkaloid accumulations occurred in matrix and margin plants in both years, although the average matrix plant was significantly higher in alkaloids only in 2002 (Appendix S2). Highest concentrations occurred in late July and early August, during the most intense period of leaf-tier activity.

## Discussion

Pyralid and gelechiid leaf-tier abundance has been inversely related to host density at Mount St. Helens in each of the last 15 years (1993 through 2007) [33]. The same pattern has been documented for root-boring Lepidoptera, not included in this study, and for *Euxoa* cutworms in two outbreak years [31]. These guilds differ in many aspects of their biology, including the tissue they feed upon, their exposure to enemies, and in phenology, leading us to hypothesize that increasing patch density or age causes differences in plant nutritional quality that affect all of these guilds similarly. We therefore considered whether variation in leaf nutrient or quinolizidine alkaloid (QA) content might explain the inverse relationship to host density.

### Herbivore Fitness Increases with %P

We detected strong relationships between larval growth and leaf %P (and in some cases leaf N∶P), and a lack of relationship of growth to leaf %N, in all species and in both feeding experiments (Fig. 1 and 2). Growth responded positively to %P in both wild-collected leaves and in greenhouse plants under competitive conditions. The result for wild-collected plants was preserved when we compared the effect of differences in nutrients between center and margin within each time period, thereby controlling for ontogenetic shifts in growth rate and leaf P (Table 1; partial *r* ^2^ = 0.61). Virtually none of the results depended on whether analyses were conducted on molar ratios or mass percentages, nor on whether RGR or larval mass was the dependent variable. While larval growth rate is only one component of individual fitness, it is likely to be correlated with overall fitness if it results in larger adult size (and hence greater fecundity), or if faster development confers other fitness benefits. In an earlier experiment in which we raised *Euxoa* on wild-collected leaves and allowed them to pupate, we observed significantly earlier pupation and higher pupal mass in rapidly growing larvae (Pearson's *r*: *r_RGR, mass_* = 0.42; *r_RGR, days to pupation_* = −0.51; *P*<0.01 for each; Appendix S5), as well as lower mortality on leaves from matrix areas, where %P was higher (Appendix S5, Fig. 4). In the subalpine environment of the Pumice Plain rapid development is likely to be advantageous because of the short growing season, the continuous decline in food quality through the summer (Fig. 4, Appendix S2), and the relatively high risk of predation in high-density core areas [31], [32]. In the single analysis of mortality that was possible (for *Euxoa*), we also detected a significant positive effect of leaf %P on survivorship (Appendix S4). Hence, our experiments suggest that increased leaf P often increases fitness of these lupin specialists in the Pumice Plain system.

### Plant Density May Determine Distribution of Herbivory through Effects on P

Leaf P content was nearly always greater in low-density lupin patches during the two years of this study (Fig. 4) and in 2000 [39]. The negative relationship between patch density and leaf P, together with evidence for herbivore sensitivity to P, supports the hypothesis that density-related differences in leaf P stoichiometry are a likely cause of the inverse relationship between lupin density and herbivore abundance. The evidence for a relationship between density, P, and larval growth is further supported by our experimental manipulations of soil P and competition in the field and greenhouse. In the greenhouse, growth was sensitive to N∶P only under competitive conditions (Fig. 2), and there was a significant positive effect of %P even after accounting for N∶P. The mechanism by which plant competition affects larval sensitivity to P and N∶P is unclear and could be related to differences in plant defense or water relations, but in any case the results suggest that P stoichiometry is likely to determine herbivore success under the high-density conditions found in Pumice Plain core areas. Likewise, in the field under high-density conditions, removal of plant competitors enhanced larval growth and survival relative to the control (Fig. 3), and indeed survivorship was ∼0 for larvae introduced to control plants. Our observations of ant, spider, and bird behavior at these sites suggests that competitor removal did not deter predator foraging on plants with a 10 cm removal zone. While the result of this experiment does not provide evidence for the role of P, it does demonstrate a strong effect of plant density on larval fitness and the potential for plant quality to exclude larvae from high-density areas.

It is plausible that effects we have attributed to P could be due to unmeasured factors, such as plant defense, that are tightly correlated with P, or to the stoichiometry of P with respect to unmeasured factors. However, differences in quinolizidine alkaloids (QAs), the principal defensive chemicals produced by lupins [47], [48], are unlikely to explain density-related patterns of herbivory in this system or the relationship of growth to P. While QAs had a significant negative effect in some regression models, neither QAs or their effect were significantly correlated with P and total QAs were either higher or equivalent in matrix and margin plants, where most feeding occurs (Appendix S2). In any case, *L. lepidus* appears to invest relatively little in QAs, as its total QAs were 1/10 or less than levels typically seen in other *Lupinus* species [49], [50]. Such a low level of defense investment is consistent with other aspects of *L. lepidus'* highly colonizing life history.

Successional dynamics underlie the gradient in plant P across high- and low-density areas of lupin. High-density vegetation patches that include a high density of lupin have developed around the oldest colonization foci. Related to their greater age, they harbor higher plant diversity, more developed soils with greater soil organismal activity, as well as more diverse and dense assemblages of vertebrates and arthropods. Surprisingly, soil N and P availability are similar between the high-density centers of core patches and the low-density margin and matrix areas [35], [39], [40], suggesting that increased plant competition or immobilization in other pools in center areas is responsible for lower nutrient levels in center plants. The onset of competitive interactions may push plant C∶P or N∶P ratios above a threshold elemental ratio that can be tolerated by herbivores, paradoxically protecting lupins from the demographic impacts of herbivores while lupin growth and fecundity become P-limited. Models of a lupin-herbivore co-invasion suggest that if herbivory in low-density margins is intense and fecundity at high density is low enough, the patch may permanently collapse, whereas at higher fecundity center areas act as demographic refugia, and spatial collapse is only temporary or may not occur [33]. Thus, P availability may influence the demography of colonizing lupins through multiple, antagonistically acting pathways whose balance determines the outcome of the co-invasion. Primary successional landscapes commonly exhibit such heterogeneity in nutrient stoichiometry as soil and community development accelerates in or spreads from foci created by initial plant colonists, and it is possible that these simple gradients promote complex spatial dynamics in colonizing populations.

### The Paradox of Enrichment

The relationship between host density, nutrient stoichiometry, and herbivore abundance is particularly interesting in light of recent extensions of Lotka-Volterra predator-prey models that explicitly incorporate stoichiometric food quality. Unlike models based only on resource quantity, the stoichiometric model demonstrates that an autotroph population may reach high biomass, thereby providing large quantities of food for potential herbivores, and yet may remain uninvaded by herbivores if high density results in low stoichiometric food quality [51]. The existence of such systems has been demonstrated experimentally in freshwater, but not in any terrestrial system [51]. Our results support a similar dynamic at Mount St. Helens, where lupin's specialist herbivores fail to exploit older patches containing high lupin biomass because plants at high density possess an unfavorable P stoichiometry. However, at Mount St. Helens this effect is likely exacerbated by higher predation risk in high-density patches.

Because insect herbivores are richer in both N and P than are autotrophs, stoichiometric studies have largely focused on the deleterious consequences of ingesting excess C in order to obtain sufficient N or P. However, studies of orthopterans feeding on non-optimal foods clearly demonstrate that there is a cost to ingesting excess protein as well as excess carbohydrates [52], and studies of aquatic herbivores [53] and two lepidopteran herbivores [23], [27] demonstrate a cost to ingesting excess P. As a result, larvae may experience declines in fitness at both high and low C∶N, C∶P, or N∶P [53]. Our greenhouse experiment provides one of the few examples of a quadratic relationship between growth and N∶P. However, as discussed above, this occurred only under competitive growing conditions, suggesting that the effect is unlikely to be caused by N∶P stoichiometry alone. In contrast to some previous studies that employed artificial diets containing unrealistic nutrient concentrations and ratios, our experiment allowed larvae to feed freely on entire plants grown under reasonably realistic conditions. Lacking still are experiments that isolate the effects of P ratios from other factors while employing realistic dietary conditions.

### N-Fixation, P-Uptake, and Herbivory on Legumes

Several other recent studies in natural systems demonstrate that enhanced P supply or leaf P concentration can affect insect herbivore population dynamics. For example, Schade et al. [25] found that the abundance of a leaf-feeding weevil on mesquite trees increased with decreasing leaf C∶P (caused by increased soil moisture). Campo and Dirzo [54], working in a secondary tropical forest found that P addition significantly increased foliage P and herbivory in leguminous trees growing in young P-limited sites, but not in older, less P-limited sites. As in the present study, these studies featured legumes as hosts. It has been suggested that herbivory is especially high on plants with N-fixing symbionts because they possess higher protein concentrations [55]. In fact, *L. lepidus* at Mount St. Helens are relatively rich not only in N but also in P, despite growing in nutrient-poor soil conditions. Mean *L. lepidus* C∶N on August 1 was nearly half the median C∶N reported by Elser et al. [17] for a sample of 406 terrestrial plants, and lupin C∶P was 65–80% of the median C∶P, making *L. lepidus* seem an unlikely host on which to find nutrient limitation. In response to P-deficient soils, other *Lupinus spp.* are known to secrete large quantities of carboxylates and phosphatase in order to obtain mineral-bound phosphate [56]. It is thus plausible that it is not the ability of legumes to symbiotically obtain atmospheric N, *per se*, that confers their ability to colonize poor soils or their high palatability to consumers, but rather an ability to extract P from P-deficient soils [57]. On the other hand, from a stoichiometric viewpoint, herbivores feeding on legumes may have a particular difficulty in obtaining sufficient P because increased P supply fuels increased photosynthesis and N-fixation, resulting in higher N∶P, C∶P, or defense:P, and concomitantly a higher cost to the herbivore of obtaining sufficient P. It remains unclear where insect herbivores are most likely to be P-limited, but systems dominated by plants with N-fixing symbioses and with low P supply are probably good candidates for this phenomenon.

## Supporting Information

Appendix S1 Images of herbivores, experiments, and representative center, margin, and matrix sites.
(19.94 MB DOC)

Appendix S2 Description of methods and results for alkaloid analysis and alkaloid induction experiment. Includes comparisons between center and matrix sites of quinolizidine alkaloid content in 2002 and 2003, and results of field induction experiment.
(0.05 MB DOC)

Appendix S3 Supplementary regression analyses: analysis of individual guilds, and of molar nutrient ratios.
(0.06 MB DOC)

Appendix S4 Regression analyses of larval mass as a function of leaf nutrients, alkaloids, and date, and analysis of *Euxoa* mortality in relation to %P. Includes mortality per day in relation to %P.
(0.07 MB DOC)

Appendix S5 Results from 2002 *Euxoa* experiment, including pupal mass and date of pupation in relation to larval RGR, and larval mortality on center vs. matrix material.
(0.04 MB DOC)
