# Supplementary material for: Successional Change in Phosphorus Stoichiometry Explains the Inverse Relationship between Herbivory and Lupin Density on Mount St. Helens
Source: PLoS One. 2009 Nov 12;4(11):e7807. doi: 10.1371/journal.pone.0007807 (PMC2771767; doi:10.1371/journal.pone.0007807)

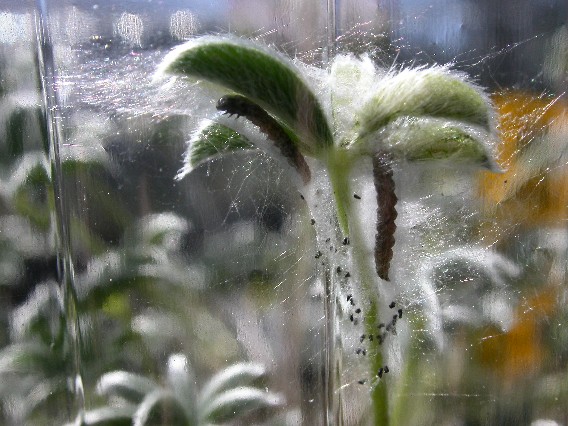

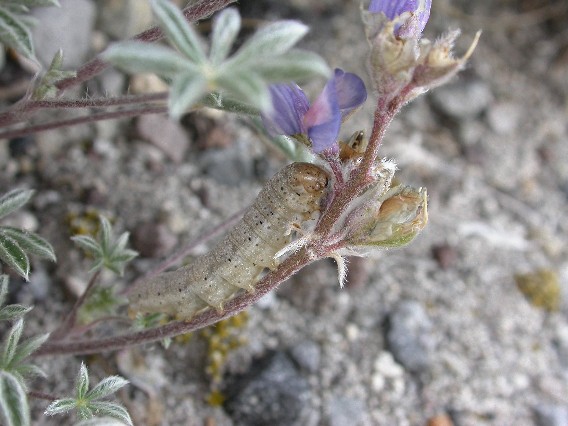
**Appendix S1.** Images of herbivores, experiments, and site types.

**Fig. S1a.** *Filatima* *loowita* (Gelechiidae) leaf tiers on *Lupinus lepidus* in greenhouse.

**Fig. S1b.** *Euxoa extranea* (Noctuidae) on *Lupinus lepidus* at Mount St. Helens.

**Fig. S1c and S1d.** Greenhouse experiment manipulating nutrient supply and grass competitors. In Fig S1c, note the damage by early instar leaf miners on lupin leaflets.


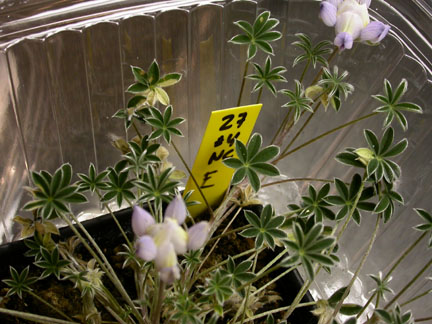


**Fig. S1d.**


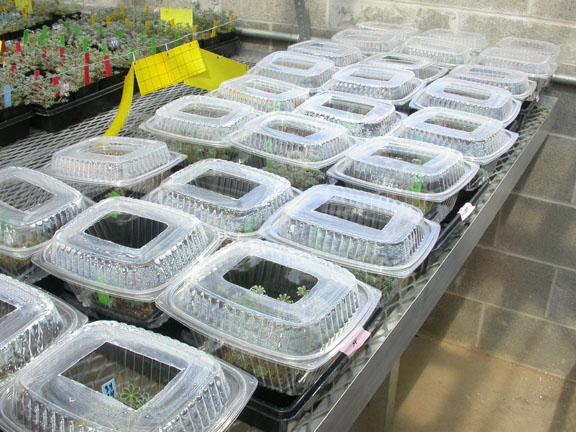


**Fig. S1e.** Format for feeding field-collected shoots to leaf-tiers and *Euxoa*.


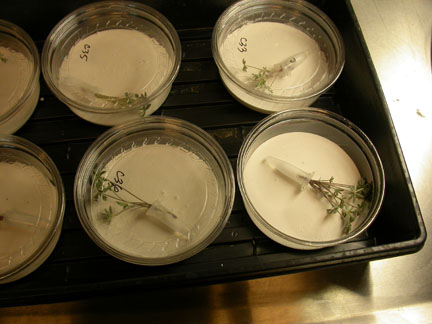


**Fig. S1f.**  Representative area of low density matrix. More than 75% of the photosynthetic area of this plant has been consumed by leaf tiers. The frame is 0.5m x 0.5m.


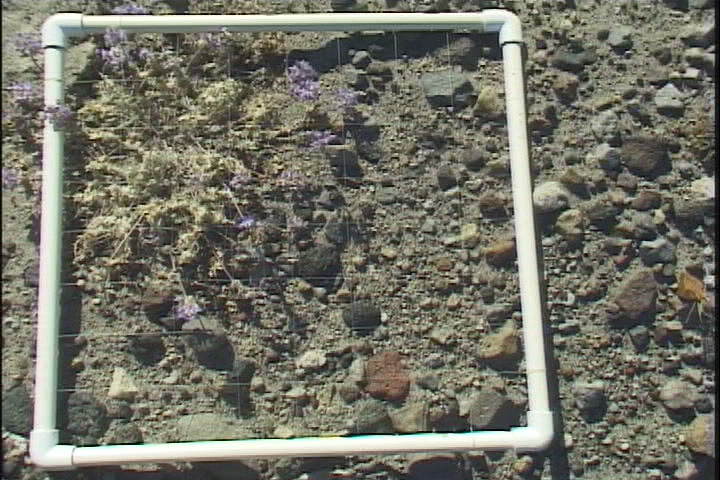


**Fig. S1g.** Representative area of high lupin density in center of core area. The frame is 0.5m x 0.5m.

**
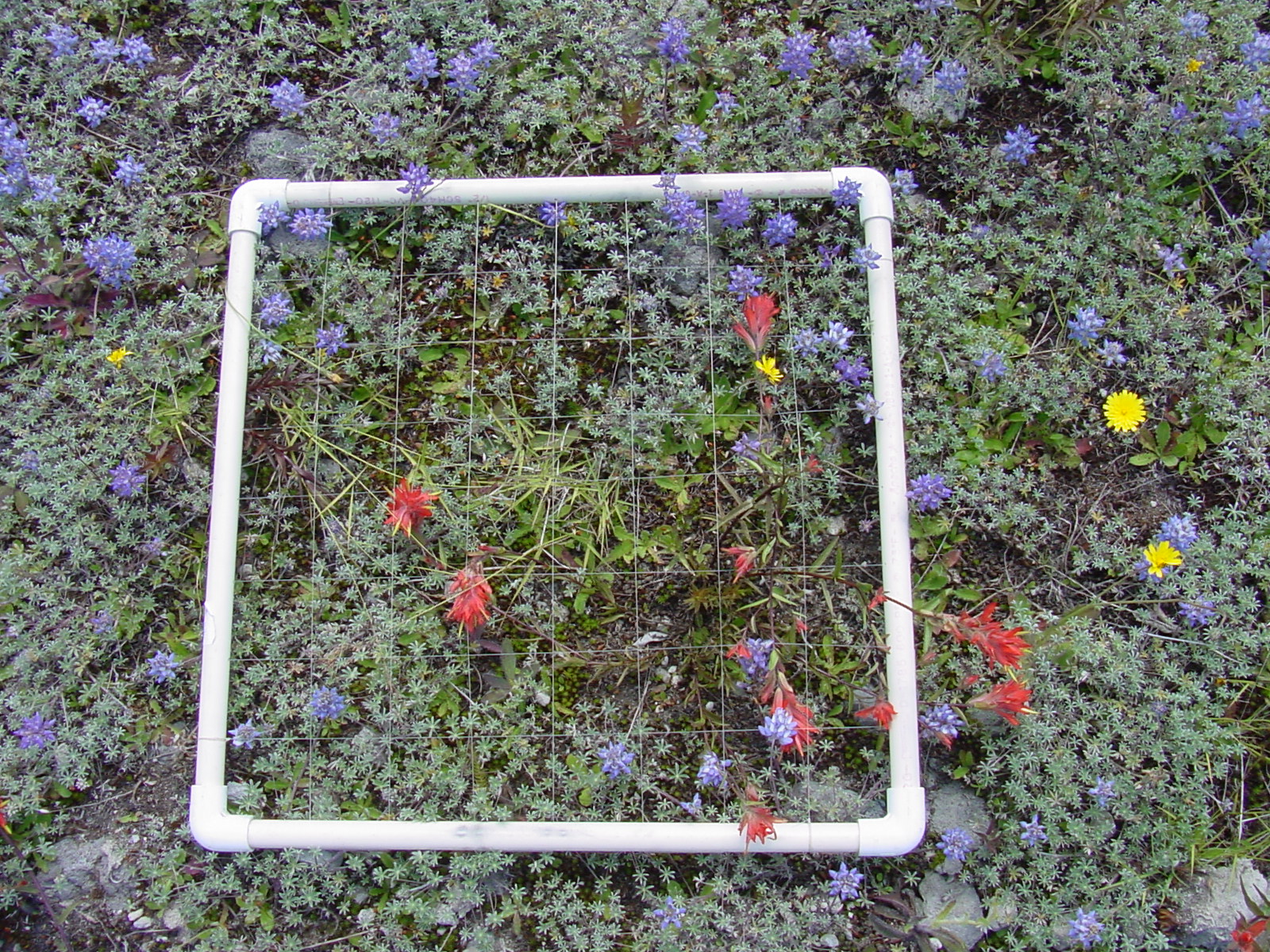
**

**Fig. S1h** Center of core area early in growing season. Plants are 10-20cm across.


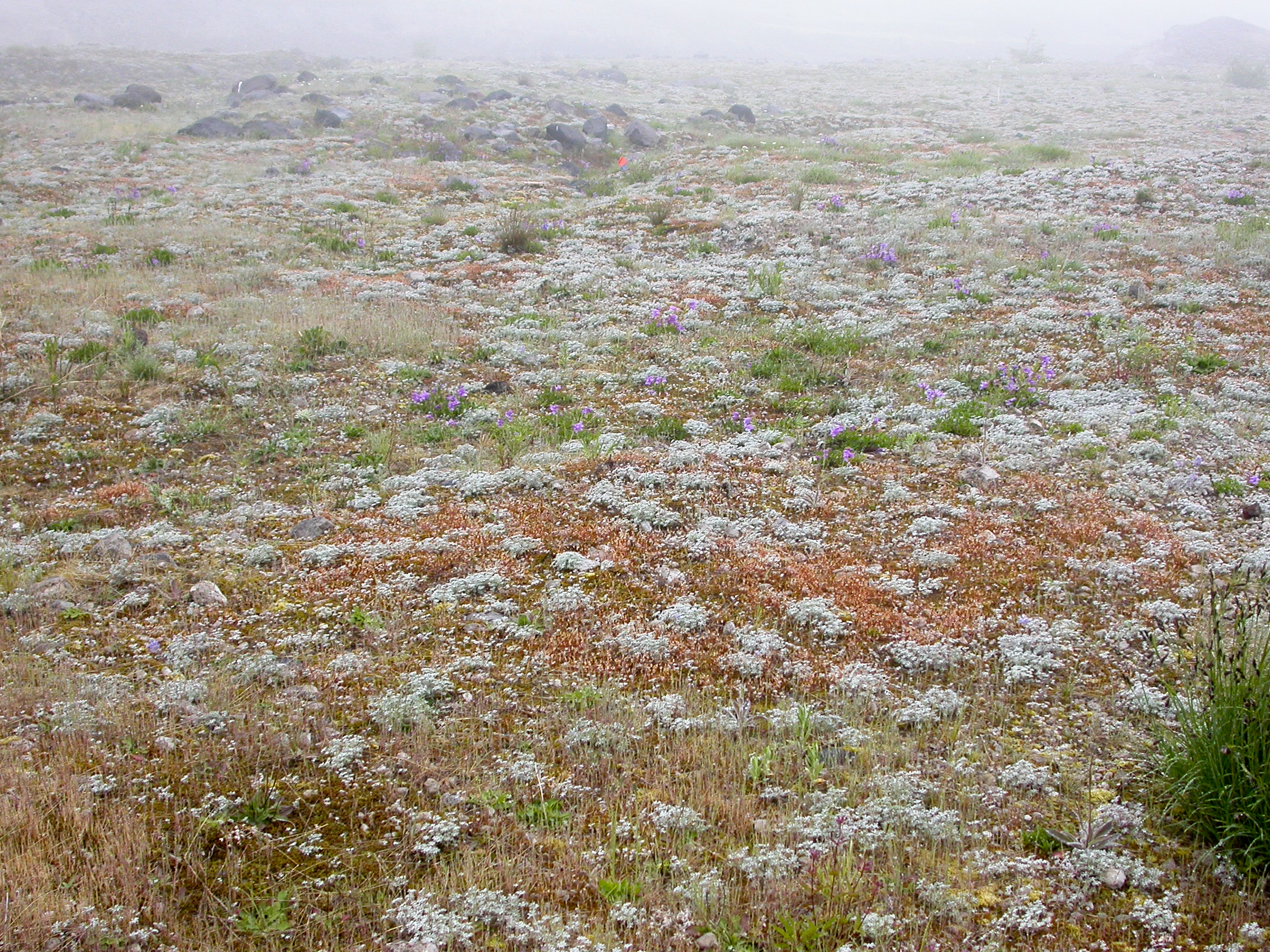


**Fig. S1i.** *Lupinus lepidus* colonizing margin of core area, early in growing season. Plants are 10-20cm across.


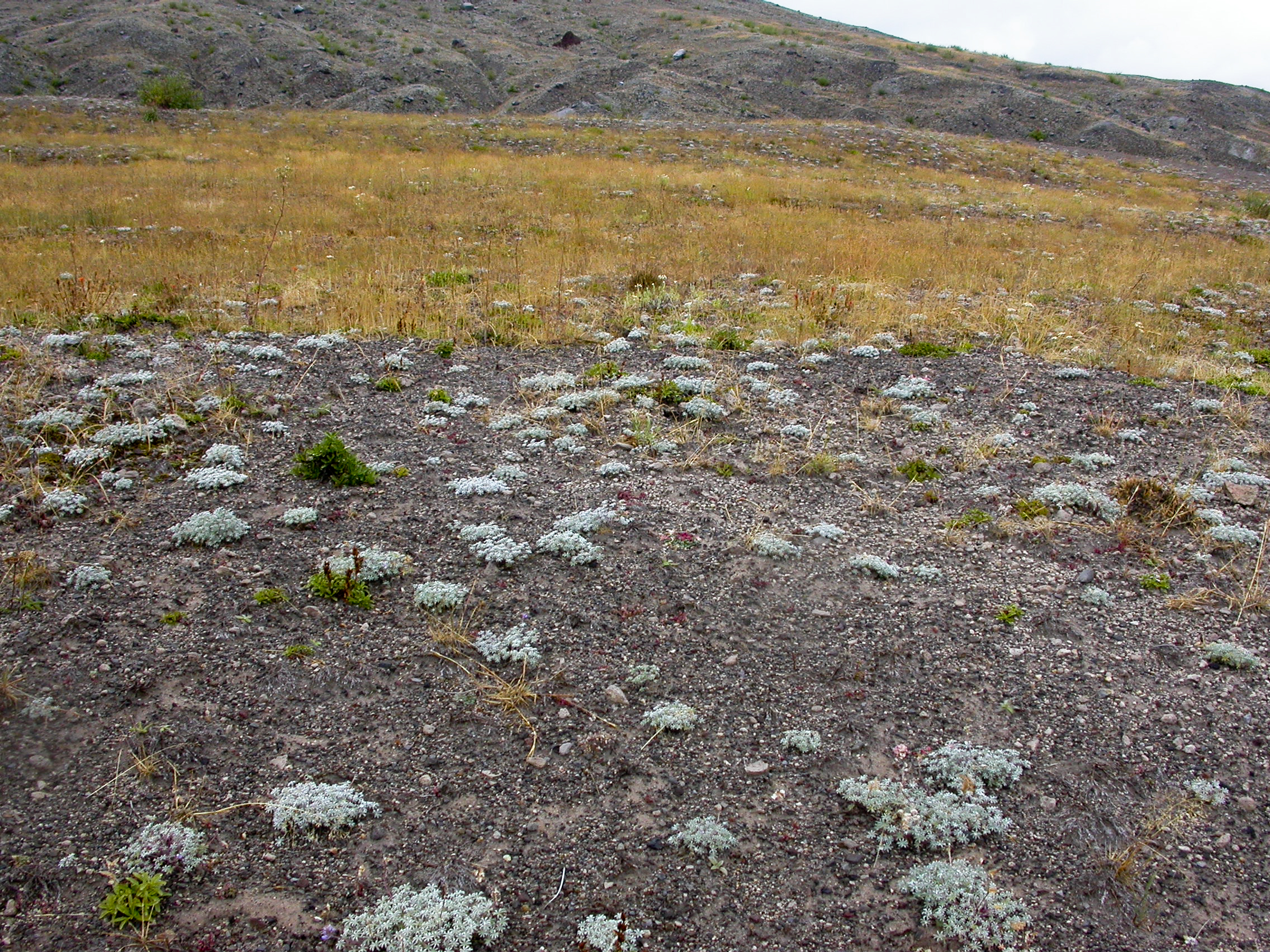


**Fig. S1j*.*** *Lupinus lepidus* colonizing low-density matrix area. Plants in foreground are ~30cm across, mesh cages are 1m tall, and distance to lake is ~4km.


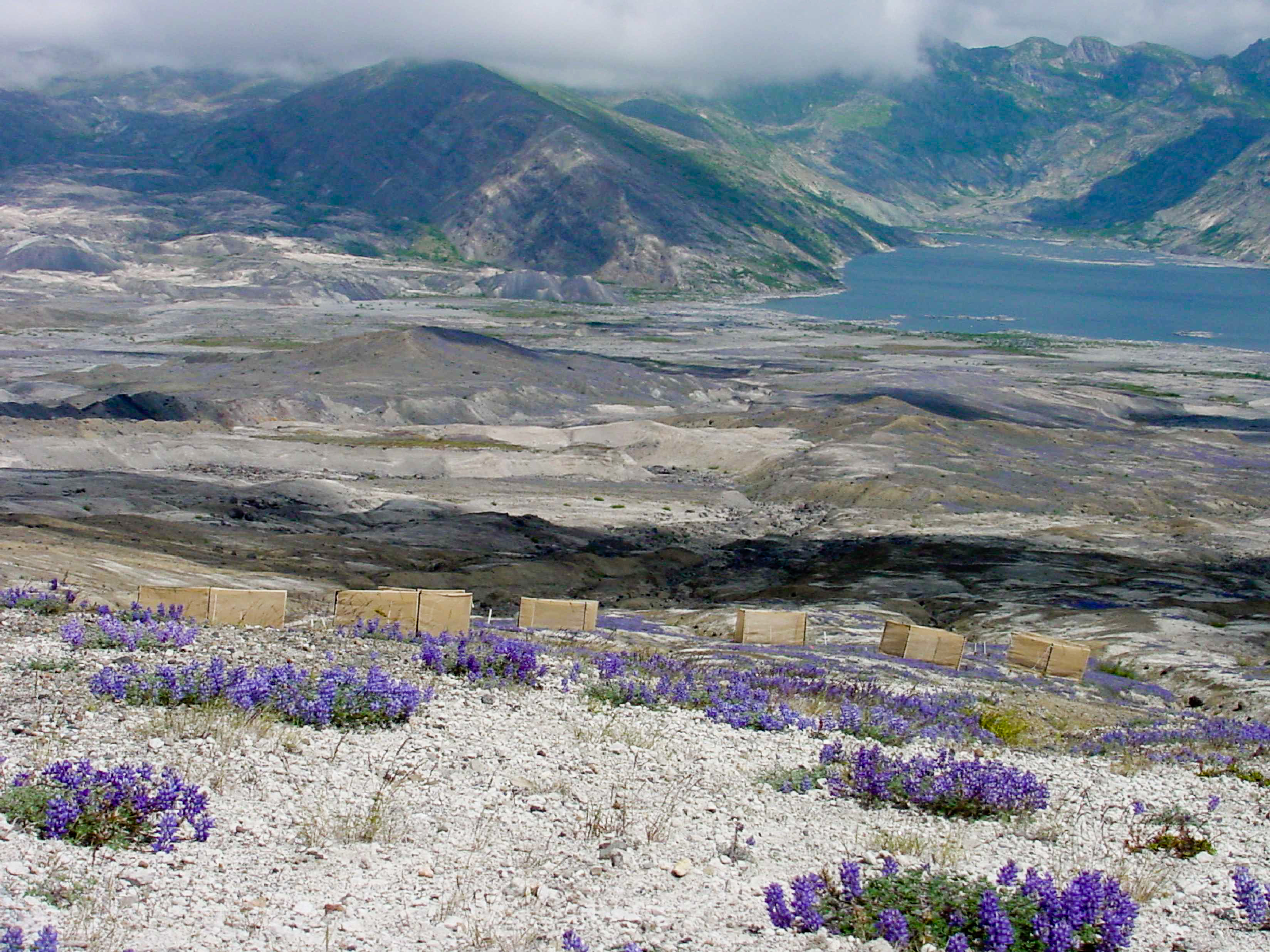


**Fig S1k.** Low-density matrix area not yet colonized by lupin. Mount St. Helens crater in background.


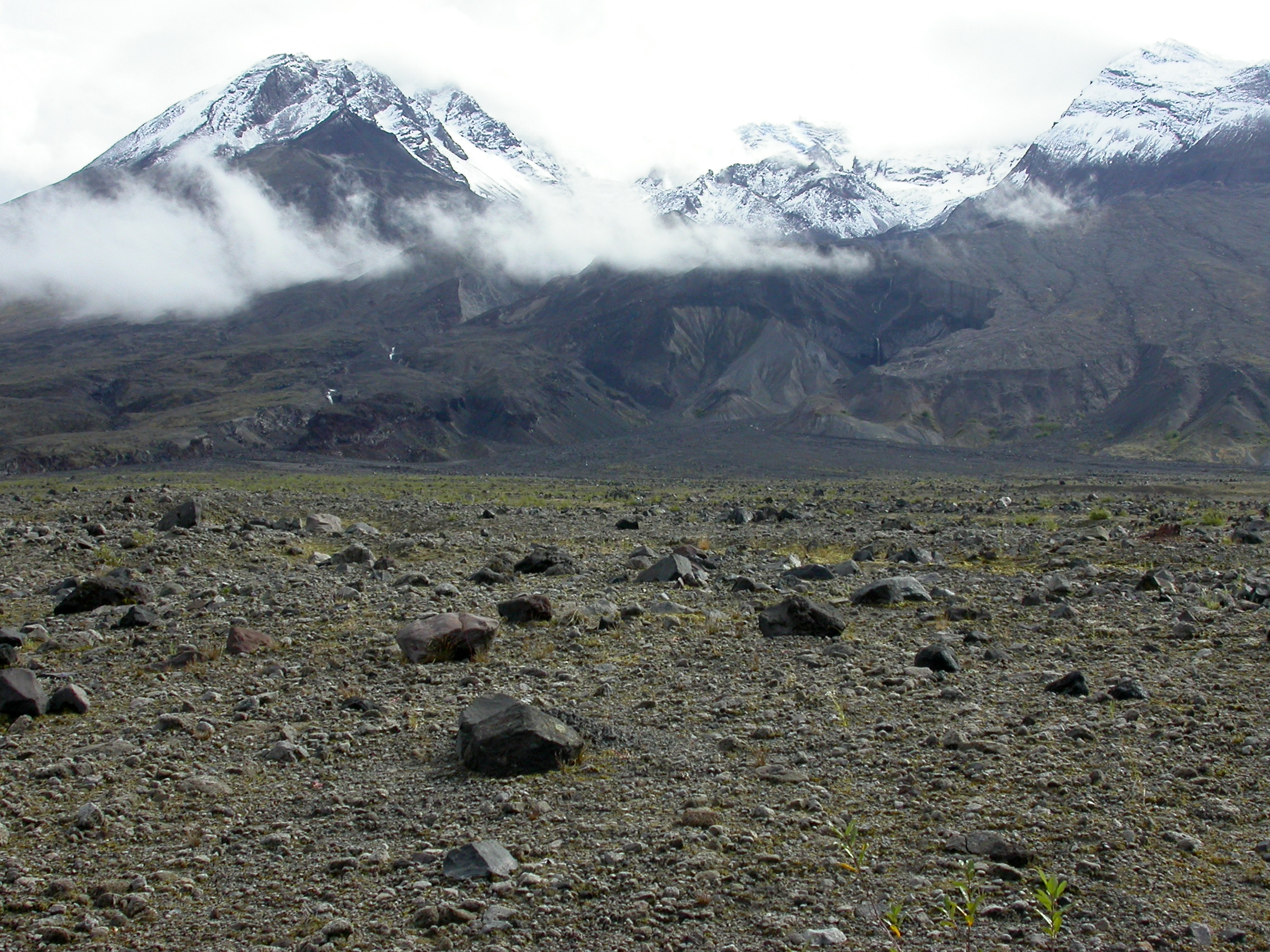


**Fig. S1l.** High-density core patch exhibiting a center area with >70% lupin cover and a low-density margin.


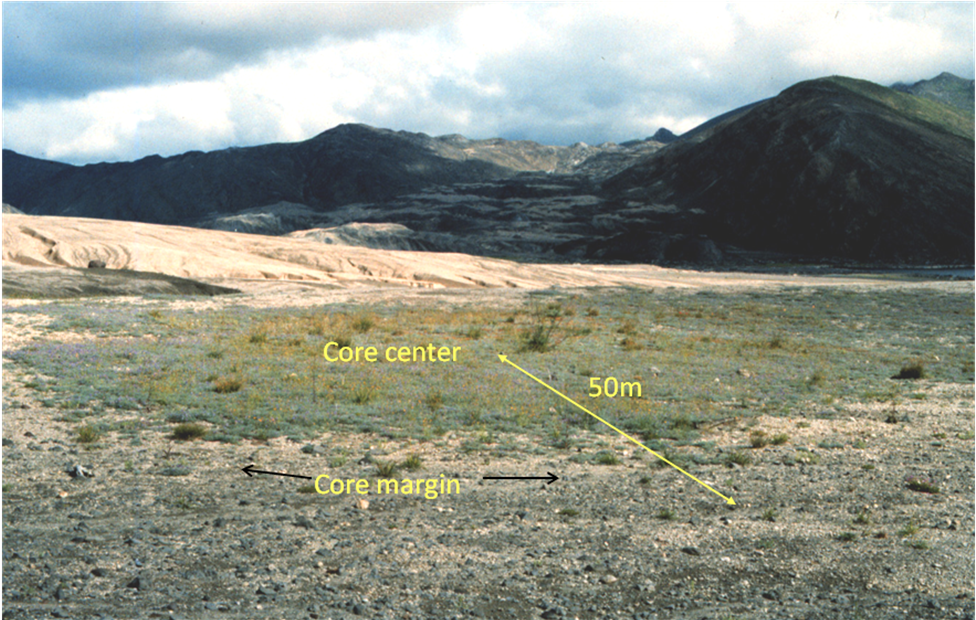


**Fig. S1m.** Field fertilization and competition experiment. This plant has had neighboring moss and vascular plants removed in a 10cm zone around the plant. 6 larvae of *Filatima loowita* were added and caused the light colored damage to leaves.


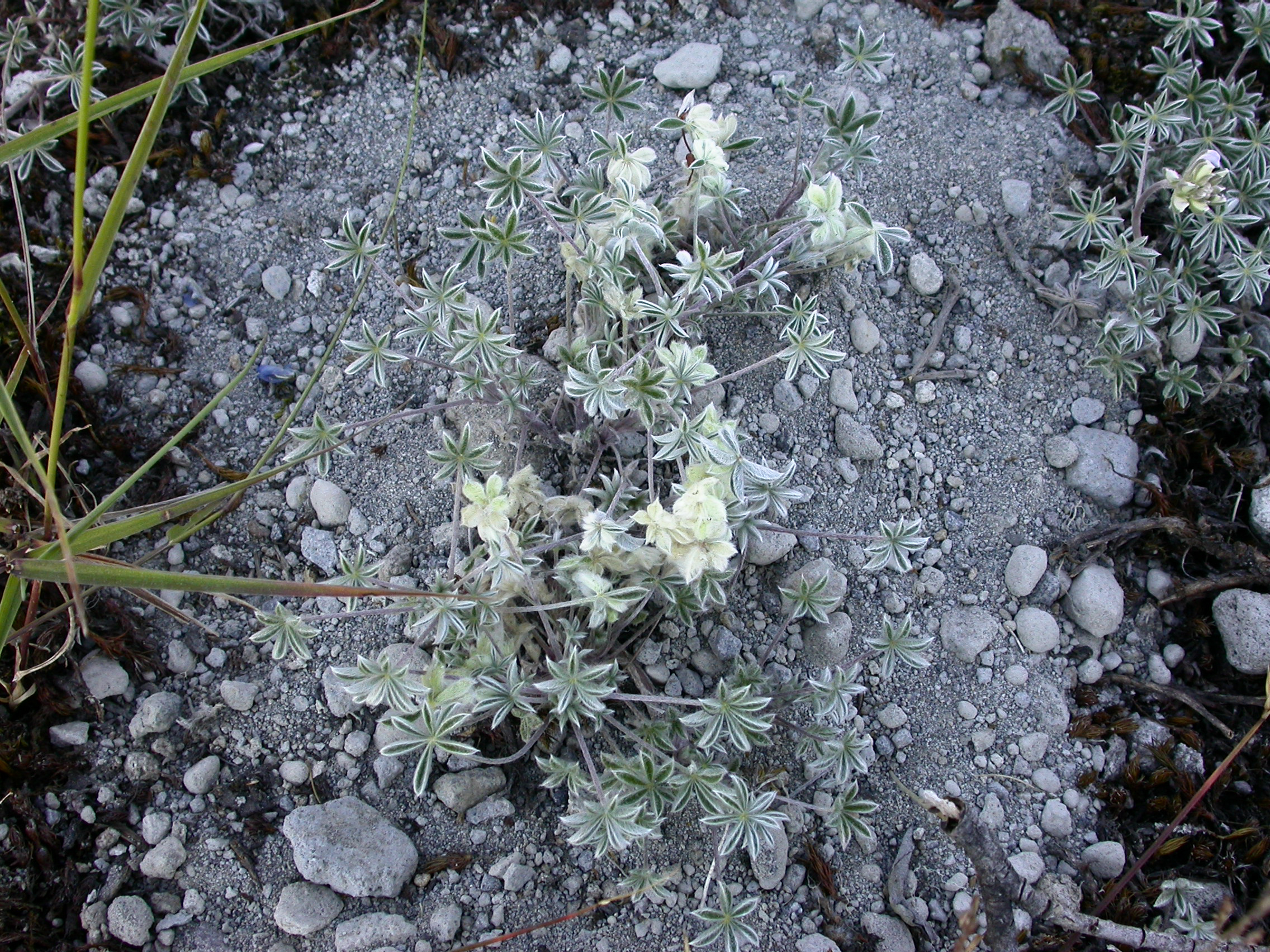

Supplement: Appendix S1 — Images of herbivores, experiments, and representative center, margin, and matrix sites. (19.94 MB DOC) [file pone.0007807.s001.doc]
