# Supplementary material for: Successional Change in Phosphorus Stoichiometry Explains the Inverse Relationship between Herbivory and Lupin Density on Mount St. Helens
Source: PLoS One. 2009 Nov 12;4(11):e7807. doi: 10.1371/journal.pone.0007807 (PMC2771767; doi:10.1371/journal.pone.0007807)
Supplement: Appendix S2 — Description of methods and results for alkaloid analysis and alkaloid induction experiment. Includes comparisons between center and matrix sites of quinolizidine alkaloid content in 2002 and 2003, and results of field induction experiment. (0.05 MB DOC) [file pone.0007807.s002.doc]

**Appendix S2. Alkaloid analysis and alkaloid induction experimental methods**

Alkaloid extraction and analysis were carried out according to Woldemichael and Wink (2002). The plant material was homogenized in 1 M HCl and left standing for 5 h. The extracts were then made alkaline with 6 M NaOH and applied onto Chemelute columns (Varian, Inc., Palo Alto, CA, USA). The free alkaloid bases were eluted with CH_2_Cl_2_. After evaporation of the solvents, the alkaloids were dissolved in 100 µL methanol and stored at 4°C. A volume of c. 1 µL was employed for high-resolution gas–liquid chromatography (GLC) and GLC-mass spectrometry (MS) for identification. GLC-MS was carried out on a Thermo-Finnigan instrument (SSQ 7000, Thermo-Finnigan, Bremen, Germany) combined with a Hewlett-Packard gas chromatograph (GC 5890 II, Hewlett-PackardGmbH, Bad Homburg, Germany). Column: 30 m × 0.25 mm; OV-1; carrier gas: helium (14 psi); temperatures: injector, 250°C; detector, 300°C; GLC program, 120 °C for 2 min, increase by 8 °C/min to 300 °C, then 6 min isothermal. MS: filament emission current, 200 mA; electron energy, 70 eV; ion source, 175°C; mass range, 60–650; scan time, 0.5 s. Alkaloids were identified according to reference alkaloids previously recorded in our lab (Wink et al. 1995). Lupanine was used as an external reference for calibration. Each measurement was performed on two samples from each bulk sample of leaf material.

To examine induction of alkaloids, two pairs of 10 m × 10 m plots were established (one pair in low-density matrix, the other in low-density margin), and one of each pair was treated with S-fenvalerate in September 2002 and June 2003 as described previously (Fagan and Bishop 2000), while controls were treated with an equivalent amount of water. This treatment effectively removed aboveground-feeding insects, though some re-colonization and below-ground herbivory occurred. Leaves were collected from multiple plants on 18 July, 19 August, and 12 September 2003 and two samples of each homogenized bulk collection were analyzed and averaged

**References**

Fagan, W. F., and J. G. Bishop. 2000. Trophic interactions during primary succession: Herbivores slow a plant reinvasion at Mount St. Helens. American Naturalist **155**:238-251.

Wink, M., C. Meissner, and L. Witte. 1995. Patterns of quinolizidine alkaloids in 56 species of the genus *Lupinus*. Phytochemistry **38**:139-153.

Woldemichael, G. M., and M. Wink. 2002. Concomitant occurrence of pyrrolizidine and quinolizidine alkaloids in the hemiparasite *Osyris alba* L. (Santalaceae). Biochemical Systematics and Ecology **30**:139-149.

**Appendix S2**

**Fig. S2a. Total leaf quinolizidine alkaloids as a percentage of dry mass in 2002 (a) and 2003 (b). (c) Alkaloid levels in herbivore removal vs. control plots in 2003. Each point is the mean of two samples of a homogenized collection of leaves from many plants.**

Center

Matrix

Center

Matrix

Center

Matrix

Center

Matrix

Center

Matrix

Center

Matrix

Center

Matrix
