# Supplementary material for: Successional Change in Phosphorus Stoichiometry Explains the Inverse Relationship between Herbivory and Lupin Density on Mount St. Helens
Source: PLoS One. 2009 Nov 12;4(11):e7807. doi: 10.1371/journal.pone.0007807 (PMC2771767; doi:10.1371/journal.pone.0007807)
Supplement: Appendix S3 — Supplementary regression analyses: analysis of individual guilds, and of molar nutrient ratios. (0.06 MB DOC) [file pone.0007807.s003.doc]

**Appendix S3. Supplementary regression analyses: separate analysis of guilds, and analyses of molar nutrient ratios.**

Here we analyze effect of leaf molar ratios (C:N, C:P, and N:P) on RGR. We also present regression analysis of %C, %N, and %P for individual guilds (*Euxoa* and leaf-tiers).

| **Dependent variable** | **Effect** | **Mean** | **Coefficient** | ***P*-value** |
| --- | --- | --- | --- | --- |
| ***Euxoa* & leaf-tier RGR^a, b^** | Alkaloids |  | -1.347 | 0.534 |
| *N* = 18, df=1,9 | C:P | 676.8 | -0.0003 | 0.024 |
|  | C:N | 22.1 | -0.0027 | 0.520 |
| ***Euxoa* RGR^a^, ^c^** | %C | 44.6 | -0.016 | 0.068 |
| *N* = 10, DF=1,6 | %P | 0.16 | 1.56 | 0.077 |
| ***Euxoa* RGR^a, d^** | C:N | 23.6 | -0.0036 | 0.764 |
| *N* = 10, DF=1,6 | C:P | 739.1 | -0.0002 | 0.083 |
| **Leaf-tier RGR^a^** | C:N | 21.8 | -0.0141 | 0.341 |
| *N* = 8, DF=1,3 |  |  |  |  |
| **Leaf-tier RGR^a^** | C:P | 629.9 | -0.0004 | 0.081 |
| *N* = 8, DF=1,3 |  |  |  |  |
| **Leaf-tier RGR^a^** | N:P | 28.8 | -0.0105 | 0.010 |
| *N* = 8, DF=1,3 |  |  |  |  |
| **Leaf-tier RGR^a^** | Alkaloids | 0.0061 | -12.46 | 0.045 |
| *N* = 8, DF=1,3 |  |  |  |  |
| **Leaf-tier RGR^a^** | %N | 2.40 | 0.1057 | 0.567 |
| *N* = 8, DF=1,3 |  |  |  |  |
| **Leaf-tier RGR^a^** | %P | 0.19 | 1.7811 | 0.088 |
| *N* = 8, DF=1,3 |  |  |  |  |
| **Leaf-tier RGR^a^** | %C | 44.8 | -0.044 | 0.016 |
| *N* = 8, DF=1,3 |  |  |  |  |
| **Center RGR –Margin RGR (*Euxoa* & leaf-tiers)^e^** | Diff. Alkaloids | -0.0025 | -1.455 | 0.042 |
| *r^2^=0.83, N* = 9, *DF* = 1,9, P=0.02 | Diff. N:P |  | 0.0025 | 0.155 |
|  | Diff. C:P | 0.0097 | -0.0002 | 0.016 |

^a^Linear mixed effects model with group identity as a random effects grouping variable to account for repeated measures at the group level, and interval as a random effect (example R command: lme(rgr ~ CP + CN + alkaloids, random= ~ interval | group.id).

^b^Nested ANOVAs indicate that C:N and alkaloids can be dropped from this model without significantly worsening model fit. In this case, the effect of C:P is more highly significant (p=0.008, df=1,11, F=10.62).

^c^ Dropping %N did not significantly change model fit.

^d^Adding alkaloids or dropping C:N did not change model fit.

^e^ Dependent variable: Center RGR - margin RGR were regressed on group identity to control for repeated measures, and the residuals were then regressed on leaf characteristics; Independent variables: Difference between center alkaloids, N:P, or C:P and margin alkaloids, C:N, or C:P. Dropping C:N did not worsen model fit.
