# Supplementary material for: Successional Change in Phosphorus Stoichiometry Explains the Inverse Relationship between Herbivory and Lupin Density on Mount St. Helens
Source: PLoS One. 2009 Nov 12;4(11):e7807. doi: 10.1371/journal.pone.0007807 (PMC2771767; doi:10.1371/journal.pone.0007807)
Supplement: Appendix S4 — Regression analyses of larval mass as a function of leaf nutrients, alkaloids, and date, and analysis of Euxoa mortality in relation to %P. Includes mortality per day in relation to %P. (0.07 MB DOC) [file pone.0007807.s004.doc]

**Appendix S4. Analysis of mass as a substitute for RGR, and of *Euxoa* mortality**

These analyses substitute larval mass for relative growth rate as the response variable in the wild-collected leaf experiment and substitute RGR for mass for the greenhouse experiment. For normally distributed data, results are from repeated measures ANCOVA of log_e_(wet larval mass) using a linear mixed effects model. Larval ID is treated as a random effect grouping variable and Date is included as an unordered random effect.

| **Dependent variable** | **Effect** | **Coefficient** | ***P*-value** |
| --- | --- | --- | --- |
| ***Euxoa* & leaf-tier log_e_(mass)**^a^ | %C | -0.02 | 0.80 |
| 18 observations in 9 groups | %N | -0.39 | 0.13 |
|  | %P | 14.58 | 0.002 |
| ***Euxoa*** log_e_**(mass)**^a,b^ | Alkaloids | -54.1 | <0.0001 |
| 387 Observations in 82 groups | %P | 24.4 | <0.0001 |
|  | %C | -0.72 | <0.0001 |
|  | %N | -0.69 | 0.006 |
| ***Euxoa*** log_e_**(mass)**^a^ | %C | 0.06 | 0.654 |
| 10 observations in 5 groups | %N | -0.36 | 0.176 |
|  | %P | 16.03 | 0.031 |
| **GREENHOUSE EXPERIMENT** |  |  |  |
| **Gelechiid RGR** **with competitors** | N:P | 0.040 | 0.002 |
| *r*^2^ = 0.61, *F* = 8.2, *DF*=3,16, *P* < 0.002 | N:P^2^ | -0.001 | 0.001 |
|  | %P | 0.727 | 0.085 |
| **Euxoa mortality per day (2003)^c^** | %P | -1.60 | 0.015 |
| *r*^2^ = 0.54, *F* =9.5, *N*=10, *P* = 0.015 |  |  |  |
|  |  |  |  |

^a^ Guild and interval included as random effect covariates to control for repeated measures; Alkaloids had no effect and were omitted from model

^b^ Mixed effects model with Larval ID and Date as random effects, using the R formula: lme(log(mass) ~ Alkaloids +leaf_P + leaf_C + leaf_N, random= ~ Interval | Larva_ID) where mass is corrected for temperature.

^c^ Mortality was calculated as average proportion dying per day over 5 intervals corresponding to each food batch. Mortality was arc-sine square root transformed to meet normality assumptions, then regressed on temperature and average larval age (thereby controlling for repeated measures at the group level). The residuals of this regression were regressed on %P.

**Supplementary Figure S4a. Relationship of mortality rate of *Euxoa* larvae to leaf % P in wild-collected leaves.** This is an added variable plot that adjusts for effects of temperature and mean larval age (residuals from regression of each variable on rearing temperature and age, plus the mean). Each point represents the mortality rate (arcsine-square root-transformed proportion of larvae dying over each interval) for larvae fed either a center or margin diet over one of 5 intervals representing different batches of food.
