# Supplementary material for: Successional Change in Phosphorus Stoichiometry Explains the Inverse Relationship between Herbivory and Lupin Density on Mount St. Helens
Source: PLoS One. 2009 Nov 12;4(11):e7807. doi: 10.1371/journal.pone.0007807 (PMC2771767; doi:10.1371/journal.pone.0007807)
Supplement: Appendix S5 — Results from 2002 Euxoa experiment, including pupal mass and date of pupation in relation to larval RGR, and larval mortality on center vs. matrix material. (0.04 MB DOC) [file pone.0007807.s005.doc]

**Appendix S5. Relationship between growth rate and other fitness parameters in *Euxoa*.**

Adult *Euxoa* were trapped on 15 August 2002 and allowed to mate and oviposit in cages. Fifty first-instar larvae were randomly assigned to each diet treatment (center vs. low-density matrix, *N*= 100; no distinction was made between edge and margin) and placed in 0.75-oz plastic condiment cups on 27 August (= day 1). Caterpillars were fed individual leaves from center or matrix lupin. Fresh lupin was field-collected from a variety of center and matrix sites every 1-2 wks and stored at 4°C. Caterpillars were fed fresh leaves every four days and were weighed every 3-7 days starting at day 10 (6 September). Larvae were maintained in a growth chamber (10-hour day, temperature range: 16°C - 26°C; mean daily temperature = 20.17°C) until pupation. Though the experiment continued until pupation in late fall, in the field larvae would enter diapause and pupate in early fall, then re-emerge in spring to complete development and pupate.

Nutrient content of food was not measured, hence, this experiment is not included in the main text. Here we note the relationship between growth rate and two other components of fitness. Larvae with higher RGR between day 0 and day 55 pupated earlier and had a higher pupal mass, as illustrated in Figure S5a (we use day 55 because it is the measurement time closest to the length of the 2003 experiment). Larvae in the lowest quartile of RGR pupated 12.5 days later on average and weighed 16% less at pupation. The fastest growing larvae pupated in 65-70 days, while the slowest growing larvae took upwards of 90 days to pupate. Regression analyses are provided below.

In addition, we note that growth and survivorship during this experiment were significantly higher for larvae feeding on plants collected from matrix areas. By day 55 larvae feeding on matrix material were 36% heavier (Mann-Whitney *U* = 283, *P* < 0.001) than larvae fed center material and pupated 10 days earlier on average (Mann-Whitney *U* = 246, *P* = 0.004), but did not differ in mass at pupation (*t* = 0.153, *DF* = 35, *P* = 0.879). Larvae fed center material experienced much higher mortality rates over the first food intervals and equivalent rates thereafter (Tarone-Ware log-rank test, χ^2^ = 4.888, *P* = 0.027; Figure S5b).

**Supplementary Figure S5a.** Relationship of larval RGR (d^-1^) to days until pupation and mass at pupation for 2002 feeding experiment. Faster-growing larvae pupated significantly earlier (coefficient = -1011, *r ^2^*= 0.258*, N* = 37, *F* = 12.15, *P =* 0.001) and were heavier upon pupation (coefficient=3.17, *r^2^* = 0.181*, N =* 37, *F* = 7.712, *P =* 0.009).

Pupal mass (g)

Time at pupation (d)

**Figure S5b.** Survivorship of *Euxoa* larvae fed on center vs. matrix material in 2002.

Center

Matrix
